# Synergistic and additive interactions between receptor signaling networks drive the regulatory T cell *versus* T helper 17 cell fate choice

**DOI:** 10.1016/j.jbc.2021.101330

**Published:** 2021-10-21

**Authors:** Douglas S. Prado, Richard T. Cattley, Corey W. Shipman, Cassandra Happe, Mijoon Lee, William C. Boggess, Matthew L. MacDonald, William F. Hawse

**Affiliations:** 1 Department of Immunology and Center for Systems Immunology, University of Pittsburgh, Pittsburgh, Pennsylvania, USA; 2 Department of Psychiatry, University of Pittsburgh, Pittsburgh, Pennsylvania, USA; 3 Department of Chemistry and Biochemistry, University of Notre Dame, Notre Dame, Indiana, USA

**Keywords:** proteomics, T regulatory cell, Treg, Th17, T cell differentiation, phospholipid signaling, IL-6R, IL-6 receptor, ITK, interlukin-2-inducible T-cell kinase, JAK, Janus kinase, TCR, T cell receptor, TEAB, triethylammonium bicarbonate, TGF-βR, TGF-β receptor, Th17, T helper 17 cell, Treg, T regulatory cell

## Abstract

CD4+ T cells differentiate into subsets that promote immunity or minimize damage to the host. T helper 17 cells (Th17) are effector cells that function in inflammatory responses. T regulatory cells (Tregs) maintain tolerance and prevent autoimmunity by secreting immunosuppressive cytokines and expressing check point receptors. While the functions of Th17 and Treg cells are different, both cell fate trajectories require T cell receptor (TCR) and TGF-β receptor (TGF-βR) signals, and Th17 polarization requires an additional IL-6 receptor (IL-6R) signal. Utilizing high-resolution phosphoproteomics, we identified that both synergistic and additive interactions between TCR, TGF-βR, and IL-6R shape kinase signaling networks to differentially regulate key pathways during the early phase of Treg *versus* Th17 induction. Quantitative biochemical analysis revealed that CD4+ T cells integrate receptor signals *via* SMAD3, which is a mediator of TGF-βR signaling. Treg induction potentiates the formation of the canonical SMAD3/4 trimer to activate a negative feedback loop through kinases PKA and CSK to suppress TCR signaling, phosphatidylinositol metabolism, and mTOR signaling. IL-6R signaling activates STAT3 to bind SMAD3 and block formation of the SMAD3/4 trimer during the early phase of Th17 induction, which leads to elevated TCR and PI3K signaling. These data provide a biochemical mechanism by which CD4+ T cells integrate TCR, TGF-β, and IL-6 signals *via* generation of alternate SMAD3 complexes that control the development of early signaling networks to potentiate the choice of Treg *versus* Th17 cell fate.

T cells continuously circulate throughout the body, encounter many different environments, and employ multiple transmembrane receptors to sense changes in their local microenvironment. Following a stable interaction between the T cell receptor (TCR) and the antigen presented by major histocompatibility complexes on the surface of a cell, an intracellular signaling cascade engages to promote T cell activation. Additionally, the local cytokine microenvironment impacts the activation trajectory and controls CD4^+^ T cell differentiation. Ultimately, the complement of CD4^+^ T cell subsets generated orchestrates the resulting adaptive immune response. Therefore, it is essential that T cells properly integrate signals to promote immunity and maintain homeostasis.

T helper cell 17 (Th17) *versus* T regulatory (Treg) is a critical CD4^+^ T cell fate choice. Th17 cells are effector cells that function in inflammatory responses to bacteria (1) and fungal (2, 3) pathogens. Signals from the TCR, IL-6 (IL-6R), and TGF-β (TGF-βR) receptors promote the expression of the RORγt transcription factor and Th17 differentiation (4, 5, 6, 7). Dysregulated Th17 cell differentiation and activity potentiate inflammation in autoimmune diseases (8, 9, 10). In contrast to Th17 cells, Tregs function to maintain immune tolerance and prevent autoimmunity. Tregs suppress immune responses (11) by secreting the IL-10 (12, 13), TGF-β (14), and IL-35 (15, 16) immunosuppressive cytokines. Tregs also express negative regulatory cell surface receptors (*e.g.*, LAG3 and CTLA4 (17)) to downmodulate immune cell activation. Signaling through the TCR and the TGF-βR promotes Treg induction (18, 19). IL-2 activates STAT5 and TGF-β1 activates SMAD signaling pathways to promote the expression of the FOXP3 transcription factor leading to Treg induction (20).

While Treg and Th17 cells have different immune functions, both differentiation programs require signals from the TCR and TGF-βR, and Th17 differentiation requires an additional IL-6R signal (4, 7, 18, 19). TGF-β signals through its associated receptor, TGF-β receptor (TGF-βR), which is a heterotetrameric surface receptor with intracellular kinase activity for serine and threonine residues. In canonical signaling, the TGF-βR phosphorylates receptor-bound SMADs (R-SMADS), including SMAD2 or SMAD3. In T cells, SMAD3 is the primary transducer of TGF-βR signaling (21, 22). In turn, these phosphorylate R-SMADs dissociate from the receptor to form hetero or homodimers that bind SMAD4 to form trimeric complexes (23). Ultimately, SMAD trimers translocate into the nucleus to assemble into coactivator and corepressor complexes to regulate gene transcription (24). The IL-6 cytokine signals through its associated transmembrane receptor, IL-6 receptor (IL-6R) (25, 26). The complex comprised of IL-6R and IL-6 associates with GP130, which triggers the activation of Janus kinases (JAKs), including JAK2. Activated JAK phosphorylates STAT3 (27), which results in STAT3 nuclear translocation and transcriptional regulation.

Many signaling pathways and kinases reciprocally regulate Treg *versus* Th17 induction. IL-2 and STAT5 signaling pathways promote the expression of the FOXP3 transcription factor leading to Treg induction while limiting Th17 differentiation (20, 28). Conversely, the interlukin-2-inducible T-cell kinase (ITK) drives Th17 differentiation but suppresses Treg induction (29). Strong AKT and mTOR signaling drives Th17 and limits Treg induction (30, 31, 32). Our recent publications demonstrate that signals such as a weak TCR signal or TCR+TGF-βR promote PDK1 to phosphorylate AKT on T308, promoting Treg induction (33, 34, 35). Strong signals activate both PDK1 and mTORC2 to phosphorylate AKT on both T308 and S473, modulating AKT substrate specificity, and induce different downstream signaling pathways (33, 34). A comprehensive comparison between signals that drive Treg and Th17 would be valuable to identify actionable targets to reestablish a healthy Treg/TH17 balance in the context of autoimmune diseases (36, 37, 38).

Protein kinases mediate signal transduction in T cells (39). Classic approaches to measure kinase signaling, such as Western blotting, allow for multiplexing. Whereas mass-spectrometry-based phosphoproteomics site specifically and quantitatively tracks thousands of phosphorylated residues simultaneously (40). Herein, we profiled the phosphoproteomes that evolve in CD4^+^ T cells stimulated during early Treg or Th17 polarization. This analysis revealed that phosphorylation regulated multiple proteins involved with signaling and metabolism. These alterations culminated in suppressed flux through the conical TCR signaling and mTOR pathways during Treg induction. Additionally, phosphatidylinositol metabolism was differentially regulated to generate elevated PtdIns(3,4,5)P3 and AKT activation during Th17 induction. Biochemical analysis demonstrated that IL-6 and TGF-β receptor signals are integrated by forming different SMAD3 complexes. During Treg induction, signaling through the TCR and TGF-β receptors promotes formation of the SMAD3/4 complex, which in turn initiates a negative feedback loop *via* PKA to suppress TCR, PI3K, and mTORC signals. During Th17 induction, signaling through the TCR, IL-6 receptor and TGF-β receptor promotes the formation of a STAT3-SMAD3 complex, which does not bind nor activate PKA. Thus, IL-6 signaling during Th17 induction *via* phospho-STAT3 promotes increased TCR, PI3K, and mTOR signaling by blocking the formation of the SMAD3/4 complex. Taken together, this work provides a molecular basis for how signals from the TCR, TGF-βR, and IL-6R synergize to select the Treg *versus* Th17 cell fate choice.

## Results

### Murine CD4^+^ T cells encode stimulation by generating different phosphoproteomes

**Figure 1:**
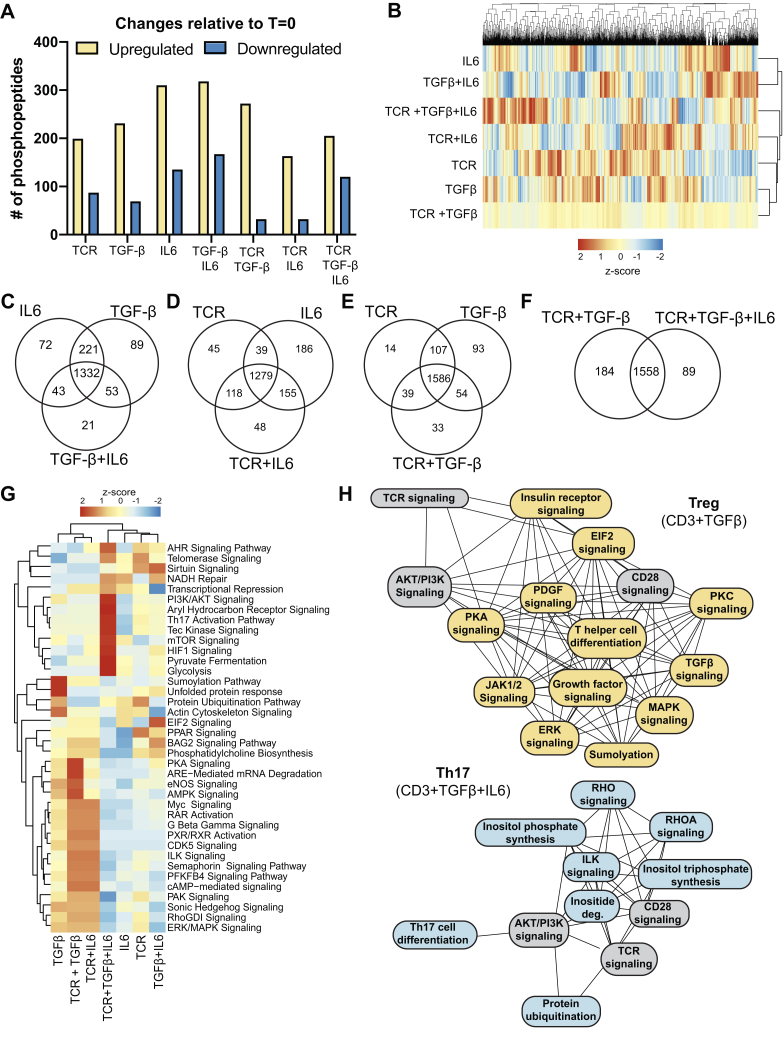
**Different combinations of TCR, TGF-β receptor, and IL-6 receptor signals generate alternate phosphoproteomes in CD4**^**+**^**T cells.** D10 CD4^+^ T cells were activated for 10 min with different TCR/CD28, TGF-β, and IL-6 stimulation combinations. A phosphoproteomic analysis measured the relative abundance of phosphorylated peptides by label-free quantitation (N = 4). *A*, phosphopeptides were grouped as upregulated or downregulated in response to stimulated compared with unactivated cells using a twofold cutoff in abundance. *B*, the relative abundance of phosphopeptides observed in CD4^+^ T cells activated with different stimuli is depicted as a heatmap. *C*–*F*, Venn diagrams were constructed to compare the common and unique phosphopeptides between CD4^+^ T cells activated with different stimuli. A twofold cutoff using the quantitative proteomics data was utilized to classify a phosphopeptide as belonging to the indicated group. *G*, the Ingenuity software package was used to identify biological pathways that were differentially targeted by protein phosphorylation across the stimulation conditions. *H*, the Ingenuity software package was utilized to identify the interconnectivity of biological pathways targeted by phosphorylation under Treg (TCR+TGF-β) *versus* Th17 (TCR+TGF-β+IL-6) stimulation conditions. A line connects pathways that share at least one common phosphoprotein. Pathways colored in *gray* are represented in both Treg or Th17 conditions, pathways unique to Treg induction are in *yellow*, and unique to Th17 induction are in *blue*.

Treg induction requires signals from TCR/CD28 and TGF-β receptors, and Th17 requires an additional signal from the IL-6 receptor. A mass spectrometric analysis characterized the phosphoproteomes generated by D10 T cells stimulated with combinations of TCR/CD28, TGF-β, and IL-6 signals 10 min postactivation (Fig. S1*A* and Table S1). On average, each activation condition yielded 2500 phosphopeptides (Fig. S1*B*). A cross-correlation analysis demonstrated high reproducibility between biological replicates (Fig. S1, *C*–*K*). The quantitative dataset contained 1831 unique phosphopeptides. On average, each stimulation condition increased the abundance of 242 phosphorylation sites (13% of the observed phosphoproteome) and reduced the abundance of 92 phosphorylation sites (5% of the observed phosphoproteome) relative to unactivated CD4^+^ T cells based on a twofold cutoff (Fig. 1*A*). The perturbation of the T cell phosphoproteome induced by the tested stimulation is consistent with previous reports that basal level phosphorylation maintains T cell homeostasis and survival (41).

There are discernable phosphorylation patterns unique to different combinations of TCR, TGF-β, and IL-6 stimuli (Fig. 1*B*). We sought to classify phosphorylation sites that were common or unique between stimuli. A twofold cutoff in abundance based on the label-free quantitative proteomic analysis was utilized. Focusing on IL-6, TGF-β, and TGF-β+IL-6 stimuli, there were 72 sites phosphorylated with IL-6 stimulation and 89 with TGF-β stimulation (Fig. 1*C*). IL-6 and TGF-β have 221 phosphorylation sites in common. Combined TGF-β+IL-6 stimulation had 43 phosphorylation sites in common with just IL-6 stimulation and 53 with just TGF-β. Combined TGF-β+IL-6 stimulation resulted in 21 unique phosphorylation sites. These data demonstrated a synergistic interaction between TGF-β and IL-6 receptor signaling networks (Fig. 1*C*).

Focusing on TCR, IL-6, and TCR+IL-6 stimuli, there were 45 phosphorylation sites unique to TCR stimulation and 186 unique to IL-6 stimulation (Fig. 1*D*). TCR and IL-6 stimulation shared 39 phosphorylation sites (Fig. 1*D*). Combined TCR+IL-6 stimulation had 118 phosphorylation sites in common with TCR stimulation and 155 in common with IL-6 stimulation (Fig. 1*D*). Combined TCR+IL-6 stimulation had 48 unique phosphorylation sites, suggesting a synergy between the TCR and IL-6 receptor signaling cascades.

The combination of TCR and TGF-β receptor signals initiates Treg induction (42). There were 14 phosphorylation sites unique to TCR stimulation, 93 unique to TGF-β stimulation, and 107 phosphorylation sites shared between TCR and TGF-β (Fig. 1*E*). The combined TCR+TGF-β stimulation condition shared 39 phosphorylation sites with TCR stimulation alone and 54 with TGF-β (Fig. 1*E*). There were 33 unique phosphorylation sites with TCR+TGF-β stimulation (Fig. 1*E*). Together, these data demonstrated that the phosphoproteome generated during Treg induction involved both additive and synergistic interactions between the TCR and TGF-β receptor networks.

TCR combined with TGF-β receptor signaling induces Treg differentiation (42). Th17 induction is induced by signaling through the TCR, TGF-β, and IL-6 receptors (4, 43). There are 1558 phosphorylation sites shared between Treg and Th17 inducing signals (Fig. 1*F*). Treg induction has 184 unique phosphorylation sites, and Th17 induction has 89 unique phosphorylation sites (Fig. 1*F*). The Treg and Th17 and phosphoproteomes have significant overlap. However, there were discernible differences in the Treg *versus* Th17 phosphoproteomes generated (14% of identified phosphopeptides).

A bioinformatic analysis identified biological pathways in CD4^+^ T cells that were differentially targeted by phosphorylation. There were diverse pathways differentially targeted, including signaling, metabolic, and RNA processing (Fig. 1*G*). To focus the analysis, we compared pathways differentially regulated by Treg (TCR+ TGF-β) *versus* Th17 (TCR+ TGF-β+IL-6) stimuli. Proteins in PKA signaling, eNOS, AMPK, and ARE mediated mRNA degradation was robustly phosphorylated during Treg induction (Fig. 1, *G* and *H*). Our previous work identified that PKA-mediated downmodulation of TCR and PI3K kinase signaling was a crucial circuit to promote Treg induction (44). AMPK signaling was also critical for Treg induction (45). Proteins in aryl hydrocarbon receptor, Tec kinase, RHO, HIF1, and inositol metabolic pathways were robustly phosphorylated during Th17 induction. Previous work identified that ITK (29), aryl hydrocarbon receptor (46), HIF1 (47), and elevated mTOR (48, 49) signaling promoted Th17 induction. These data demonstrate that kinase signaling networks are differentially engaged to control the Treg *versus* Th17 T cell fate choice.

### Treg polarization promotes phosphorylation of negative regulators to dampen TCR signaling

**Figure 2:**
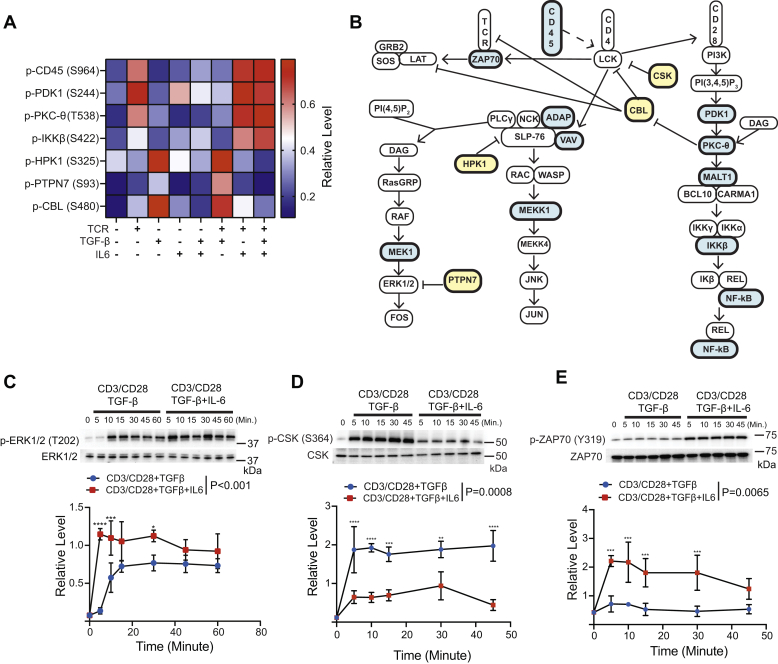
**Proteins in the TCR signaling cascade are differentially phosphorylated during Treg and Th17 induction.***A*, label-free mass spectrometry determined the relative abundance of phosphopeptides for proteins in the TCR signaling cascade. *B*, a schematic of the TCR signal transduction pathway is depicted. Proteins hyperphosphorylated under Th17 (TCR/CD28+TGF-β+IL-6) stimulation conditions are colored in *blue*. Proteins hyperphosphorylated under Treg (TCR/CD28+TGF-β) stimulation are colored in *yellow*. Immunoblotting was performed on lysates derived from primary murine CD4^+^ T cells activated under Treg or Th17 induction conditions for (*C*) p-ERK1/2 (T202/Y204), (*D*) p-CSK (S364), and (*E*) p-ZAP70 (Y319). Densitometry was performed across three biological replicates. Phosphorylated proteoforms were normalized to total protein levels. Shown are mean ± SD; *p* values were calculated by two-way ANOVA (∗∗∗∗*p* < 0.0001, ∗∗∗*p* < 0.001, ∗∗*p* < 0.01, ∗*p* < 0.1). Source data files for panels *C–E* are provided.

Proteins in the TCR signaling cascade were phosphorylated in response to both Treg (TCR+TGF-β) and Th17 (TCR+TGF-β+IL-6) stimuli (Fig. 2, *A* and *B*). The abundance of phosphorylation was dependent on the TCR, IL-6, and TGF-β signal (Fig. 2*A*). CD45 is a phosphatase that removes inhibitory phosphorylation on LCK to initiate proximal TCR signaling. Phosphorylation on CD45 S964 stimulates phosphatase activity (50). Most conditions containing a TCR signal had elevated phosphorylation of S964, except for stimulation with TCR+TGF-β, which resulted in suppressed S964 phosphorylation (Figs. 2*A* and S2). Downstream of proximal TCR signaling, PtdIns(3,4,5)P3 generation activates PDK1 marked by autophosphorylation on S244 (51). Most conditions that included a TCR stimulus resulted in elevated PDK1 S244 phosphorylation (Figs. 2*A* and S2). However, stimulation with TCR+TGF-β resulted in diminished S244 phosphorylation (Figs. 2*A* and S2). Phosphorylation of T538 on PKC-θ stimulates its kinase activity (52). T538 on PKC-θ was robustly phosphorylated in conditions that contained TCR stimulation, while TCR+TGF-β stimulation dampened phosphorylation on T538 (Figs. 2*A* and S2). Proteins downstream of PKC-θ, including IKKB, had increased phosphorylation levels relative to the TCR+TGF-β condition (Figs. 2*A* and S2). Other proteins involved with signaling cascades downstream of proximal TCR signaling that promote JUN and FOS activation, including ERK1/2, had increased phosphorylation in response to Th17 (TCR+TGF-β+IL-6) relative to Treg (TCR+TGF-β) stimulation (Figs. 2*C* and S2).

The phosphorylation of negative regulators of TCR signaling, including HPK1, PTPN7, and CBL, was increased during Treg (TCR+TGF-β) *versus* Th17 induction (TCR+TGF-β+IL-6) (Figs. 2*A* and S2). TGF-β stimulation also resulted in hyperphosphorylation of HPK1 and CBL, while IL-6 receptor signaling blunted phosphorylation (Figs. 2*A* and S2). The PTPN7 phosphatase downmodulates ERK1/2 phosphorylation and activation (53). T cells activated with TCR+TGF-β had reduced levels of Y204 phosphorylation on ERK1/2 *versus* TCR+TGF-β+IL-6 stimulation (Fig. 2*C*). Our previous work demonstrated that TGF-β receptor signaling activates CSK to inhibit the LCK kinase to dampen TCR signaling (44). Immunoblotting revealed that Treg polarization resulted in higher levels of p-CSK (S364) relative to Th17 polarization (Fig. 2*D*). Phosphorylation of ZAP70, which LCK catalyzes, was downmodulated during Treg polarization (Fig. 2*E*), which demonstrated elevated CSK activity during Treg compared with Th17 induction. These data identified multiple negative regulatory circuits that dampened TCR signal transduction during Treg induction.

### Signaling inputs regulate phosphatidylinositol metabolism in CD4^+^ T cells

**Figure 3:**
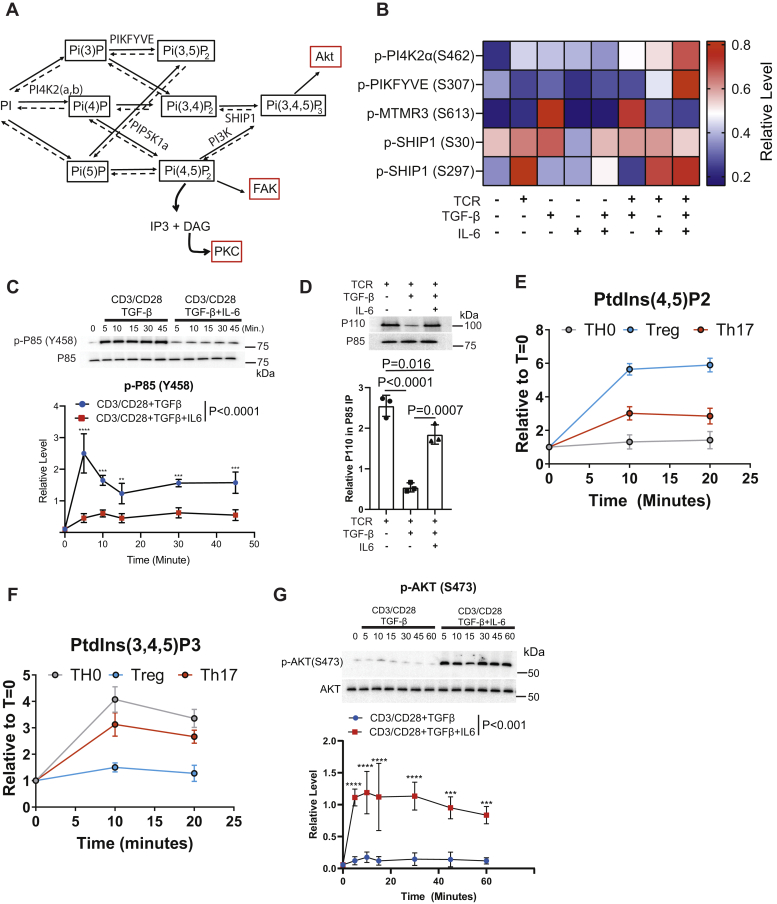
**Phosphatidylinositol metabolism is differentially regulated during Treg *versus* Th17 induction.***A*, a schematic of phosphatidylinositol metabolism is depicted. *B*, the label-free phosphoproteomic analysis determined the relative abundance of phosphopeptides for phosphatidylinositol kinases and phosphatases regulated by signaling input. *C*, primary CD4^+^ murine T cells isolated by negative selection were activated under Treg (anti-CD3 AB, soluble CD28 AB, and TGF-β) or Th17 (anti-CD3 AB, soluble anti-CD28 AB, TGF-β, and IL-6) induction conditions. Immunoblotting was performed for p-P85 (Y458) and total P85. Densitometry was performed across three biological replicates to quantitate p-P85 (Y458) normalized to total p85 levels. Shown are mean ± SD; *p* values were calculated by two-way ANOVA (∗∗∗∗*p* < 0.0001, ∗∗∗*p* < 0.001, ∗∗*p* < 0.01). *D*, primary murine CD4^+^ T cells were activated with under TH0 (anti-CD3, soluble anti-CD28 antibodies), Treg (anti-CD3 and soluble anti-CD28 antibody and TGF-β) and Th17 (anti-CD3 antibody, anti-CD28 antibody, TGF-β, and IL-6) for 10 min. P85 was immunoprecipitated. Immunoblotting was performed for P85 and P110. Densitometry was performed across three biological replicates. Each data point represents the mean ± standard deviation across three independent experiments and one-way ANOVA was performed. The abundances of (*E*) PtdIns(4,5)P2 and (*F*) PtdIns(3,4,5)P3 were measured with an imaging flow cytometry assay in primary murine CD4^+^ T cells activated under TH0, Treg, and Th17 conditions (defined above). Three biological replicates were performed. Presented is the mean ± standard deviation. *G*, primary murine CD4^+^ T cells were activated under Treg and Th17 conditions (defined above), and immunoblotting was performed on the resulting lysates for p-AKT(S473) and total AKT. Densitometry was performed on the immunoblots. Three biological replicates were included. Phosphorylated AKT was normalized to the total AKT abundance. Shown are mean ± SD; *p* values were calculated by two-way ANOVA (∗∗∗∗*p* < 0.0001, ∗∗∗*p* < 0.001). Source data are provided for the immunoblots in panels *C*, *D*, and *G*.

T cell activation promoted the phosphorylation of enzymes associated with phosphatidylinositol metabolism (Fig. 1*G*), a critical pathway regulating CD4^+^ T cell activation and differentiation. PtdIns(3,4,5)P3 generated by the lipid kinase PI3K activates AKT, a protein kinase that functions in T cell fate decisions (54) (Fig. 3*A*). Additionally, T cell activation induces the degradation of PtdIns(4,5)P2 to IP3 and DAG to promote calcium release and PKC activation.

All stimuli tested increased the phosphorylation of S462 on PI4K2a, which phosphorylates phosphatidylinositol to yield PtdIns (4)P (Figs. 3*B* and S3). The lipid kinase PIKFYVE had elevated phosphorylation on S307 levels only in response to TCR+TGF-β+IL-6 stimulation (Figs. 3*B* and S3). MTMR3 is a lipid phosphatase that had elevated phosphorylation on S613 in response to TGF-β or TCR+TGF-β stimulation, while IL-6 receptor signaling blunted MTMR3 phosphorylation (Figs. 3*B* and S3). The lipid phosphatase SHIP-1 was phosphorylated on S30 and S297 in CD4^+^ T cells (Figs. 3*B* and S3). S297 phosphorylation occurred at increased levels in stimulation conditions that contained TCR stimulation except for TCR+TGF-β stimulation (Figs. 3*B* and S3).

Previously, we found that CSK-catalyzed phosphorylation of P85 in CD4^+^ T cells disrupted the PI3K P85/P110 heterodimer, which reduced PtdIns(3,4,5)P3 biosynthesis (44). Given that CSK activity was higher in Treg induction (Fig. 2, *D* and *E*), we reasoned that P85 phosphorylation might be critical for the Treg *versus* Th17 cell fate choice. Treg polarization resulted in more robust phosphorylation of P85 than under Th17 polarization (Fig. 3*C*). P85 was immunoprecipitated from T cells activated under TH0, Treg, and Th17 conditions (Fig. 3*D*). Immunoblotting demonstrated that Treg polarization disrupted the P85-P110 heterodimer, while activation under Th17 or Th0 conditions maintained P85-P110 dimer abundance (Fig. 3*D*).

Phosphoproteomic and biochemical analysis suggested that phosphatidylinositol metabolism differs between Treg and Th17 induction (Fig. 3, *B*–*D*). An imaging flow cytometry assay measured the relative levels of PtdIns(4,5)P2 (Fig. 3*E*) and PtdIns(3,4,5)P3 (Fig. 3*F*) in primary murine CD4^+^ T cells activated under TH0 (TCR/CD28), Treg (TCR/CD28+TGF-β), or TH17 (TCR+TGF-β+IL-6) conditions. Activation under Treg conditions resulted in high PtdIns(4,5)P2 abundance, while Th17 induction resulted in intermediate, and TH0 resulted in lower abundance (Fig. 3*E*). PtdIns(3,4,5)P3 abundance was high in TH0 and Th17 activation conditions but low in Treg conditions (Fig. 3*F*). Our previous work demonstrated that high PtdIns(3,4,5)P3 levels are required to activate mTORC2 to phosphorylate S473 on AKT, and differential phosphorylation on S473 regulated AKT substrate specificity in CD4^+^ T cells (33, 44). Th17 induction generated high levels of PtdIns(3,4,5)P3 (Fig. 3*F*). Accordingly, immunoblotting demonstrated that Th17 induction caused elevated S473 phosphorylation on AKT (Fig. 3*G*). These data showed that phosphatidylinositol and AKT signaling were differentially regulated between Th17 and Treg induction.

### CD4^+^ T cells utilize the AKT/mTOR axis to discriminate between TCR, TGF-β, and IL-6 signaling inputs

**Figure 4:**
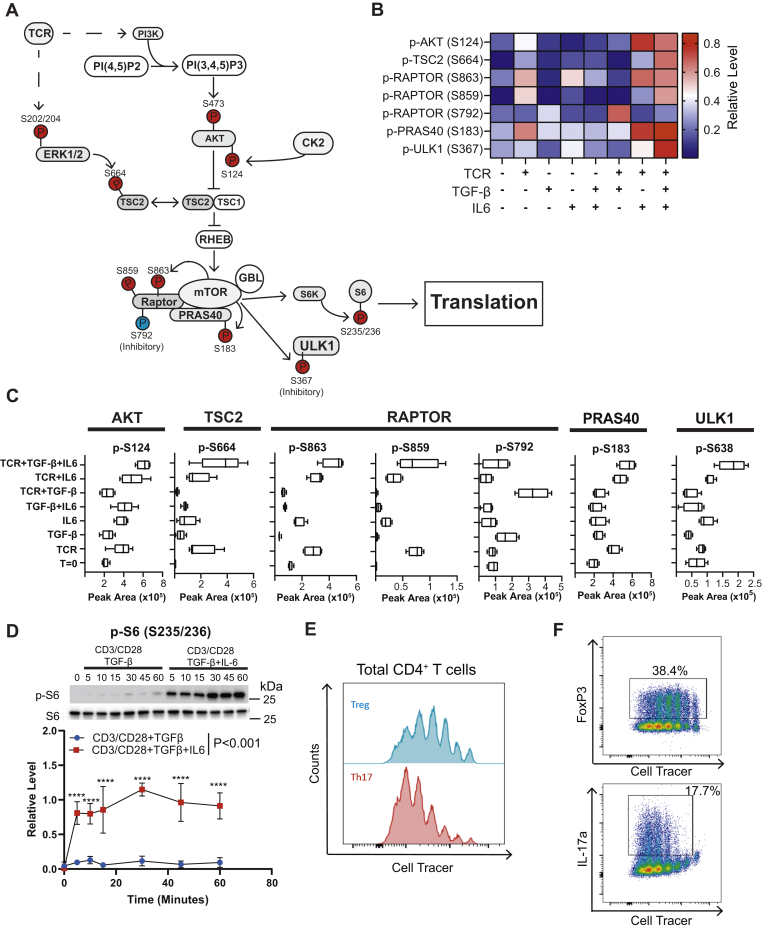
**Different stimuli result in differential phosphorylation of components in the AKT/mTOR signaling axis in murine CD4**^**+**^**T cells.***A*, depicted is a schematic of the AKT/mTOR signaling axis in T cells. Phosphorylated residues with increased abundance are highlighted in *red* for Th17 stimuli (TCR+TGF-β+IL-6) and *blue* for Treg stimuli (TCR+TGF-β), based on phosphoproteomic and biochemical data. *B*, label-free quantitative phosphoproteomics determined the relative abundance of phosphorylation on proteins associated with AKT/mTOR signaling. *C*, the peak area defined by the label-free quantitation is depicted for phosphorylation sites on AKT, TSC2, the RAPTOR subunit of mTOR, PRAS40, and ULK1 across the various stimulation conditions. In the bar and whiskers plot, the *bottom* of the bar represents the 25th percentile, the top represents the 75th percentile, and the *line within the box* represents the mean across four independent experiments. The whiskers are drawn the lowest and highest peak areas observed. *D*, primary murine CD4^+^ T cells activated under Treg (anti-CD3, soluble anti-CD28 antibodies, and TGF-β) or Th17 (anti-CD3, soluble anti-CD28 antibodies, TGF-β, and IL-6) stimulation. Immunoblotting for p-S6 (S235/236) and total S6 protein was performed. Densitometry was performed on immunoblots normalizing p-S6 (S235/236) to total S6 protein. Each data point represents the mean ± standard deviation across three independent experiments. Shown are mean ± SD; two-way ANOVA calculated *p* values (∗∗∗∗*p* < 0.0001). Primary murine naïve CD4^+^ T cells were isolated by negative selection, labeled with Cell Tracer dye, and polarized under Treg or Th17 conditions. Cells were analyzed by flow cytometry. *E*, CD4^+^ cells were gated and the cell count was plotted as a function of incorporated Cell Tracer and (*F*) for FoxP3 and IL-17a. Three biological replicates were included in the cell proliferation assays depicted in panels *E* and *F*. Source data are provided for the immunoblots in panel *D*.

The AKT/mTOR signaling axis regulates T cell growth (55), metabolic programs (56), and T cell fate decisions (35, 48). Protein phosphorylation regulates flux through the AKT/mTOR pathway (Fig. 4*A*). Most activation conditions promoted phosphorylation of S124 (Fig. 4, *B* and *C*), a known promoter of AKT enzymatic activity (57). Activation with TGF-β or TCR+TGF-β conditions however suppressed levels of p-AKT (S124). IL-6 receptor signaling restored p-AKT (S124) in T cells activated in the TCR/CD28+TGF-β-IL-6 condition, suggesting that IL-6 receptor signaling overrode TGF-β suppression and permits AKT S124 phosphorylation. The TSC1-TSC2 complex suppresses mTORC1 activation. Phosphorylation of TSC2 on S664 disrupts the TSC1-TSC2 complex promoting mTORC1 activation (58). Conditions containing TCR activation had increased p-TSC2 (S664). However, the TCR+TGF-β condition had reduced S664 phosphorylation (Fig. 4, *B* and *C*), which is an ERK1 substrate (58, 59). Therefore, increased p-TSC2 (S664) was consistent with increased ERK activation in the TCR+TGF-β+IL-6 *versus* the TCR+TGF-β condition (Fig. 2*C*).

Phosphorylation of the Raptor subunit regulates mTORC1 kinase activity (Fig. 4*A*). Phosphorylation of S863 and S859 on RAPTOR stimulates mTORC1 activity (60, 61). TCR stimulation increased S863 and S859 phosphorylation, while TGF-β receptor signaling blunted phosphorylation of these sites (Fig. 4, *B* and *C*). Phosphorylation of S792 on RAPTOR inhibits mTORC1 activity (62). Stimulation with TGF-β or TCR+TGF-β promoted phosphorylation of RAPTOR S792, while IL-6 receptor signaling blunted RAPTOR S792 phosphorylation (Fig. 4, *B* and *C*). These data demonstrated that CD4^+^ T cells integrated TCR/CD28, TGFβR, and IL-6R signals by generating alternate phosphorylation patterns on the RAPTOR subunit of mTORC1.

Proteins downstream of mTORC1 were differentially regulated by TCR, TGF-βR, and IL-6R signals. PRAS40 is a component of the mTORC1 complex and is directly phosphorylated on S183 by mTOR (63). TCR stimulation elevated levels of p-PRAS40 (S183), while TCR+TGF-β signaling suppressed p-PRAS40 (S183) (Fig. 4, *B* and *C*). MTORC1 catalyzes the inhibitory phosphorylation of S638 on ULK1. TCR or IL-6 single stimulation increased p-ULK1 (S638) (Fig. 4, *B* and *C*). Combined TCR+IL-6 stimulation and TCR+IL-6+TGF-β had increased p-ULK1 (S638), while TCR+TGF-β had suppressed levels of p-ULK1 (S638). A final marker studied was the phosphorylation of S6 *via* S6 kinase, downstream from mTORC1 activation. Primary murine CD4^+^ T cells were activated under Treg (TCR+TGF-β) or TH17 (TCR+TGF-β+IL-6) conditions. Immunoblotting revealed that p-S6 (S235/S236) was more abundant during Th17 than Treg induction (Fig. 4*D*).

The mTOR pathway promotes cell growth and proliferation (64, 65). Based on the proteomic data, the flux through the AKT/mTOR pathway is suppressed during Treg *versus* Th17 differentiation (Fig. 4, *B* and *C*). Cell proliferation assays were performed on naïve CD4^+^ T cells differentiated under Treg or Th17 polarization conditions (Fig. 4, *E* and *F*). In analyzing CD4^+^ T cells, cells were less proliferative under Treg compared with Th17 conditions (Fig. 4*E*). FoxP3^+^ CD4^+^ T cells also developed in cells that underwent fewer cell divisions, whereas IL-17^+^CD4^+^ T cells preferentially developed in cells that underwent more cell divisions (Fig. 4*F*). Our data provide a mechanism by which kinase signaling networks regulate the flux through the AKT/mTORC1 signaling axis between the Treg and Th17 cell fate choice.

### CD4^+^ T cells integrate TGF-β and IL-6 receptor signals by generating different SMAD3 complexes

**Figure 5:**
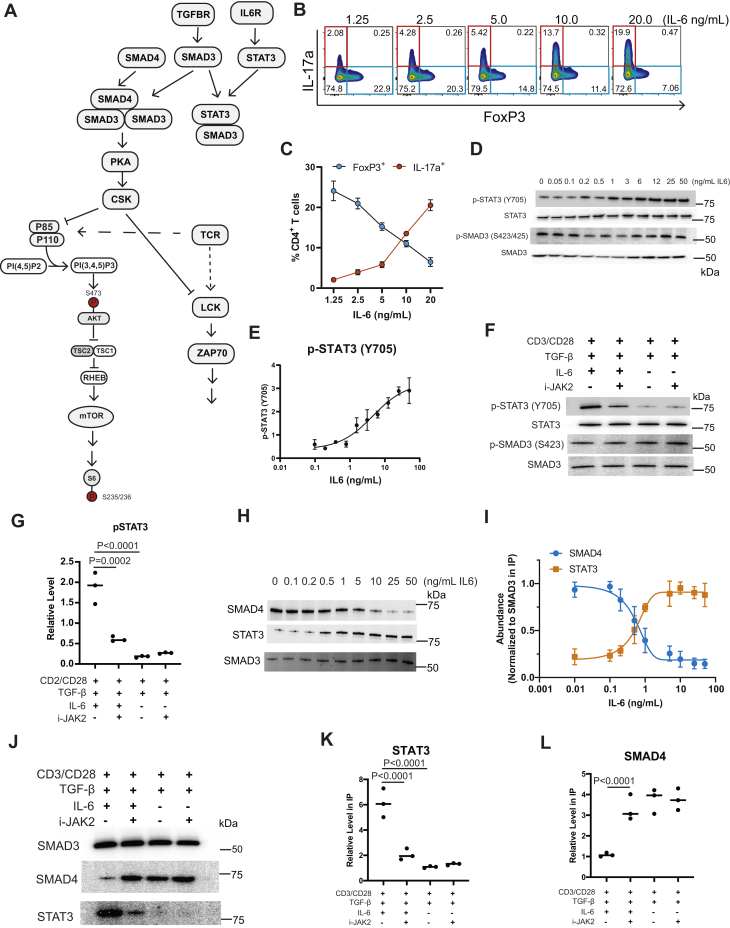
**CD4**^**+**^**T cells integrate TGF-β and IL-6 receptor signals by forming different SMAD3 complexes.***A*, depicted is a minimized model of TCR, IL-6R, and TGF-βR signaling in a CD4^+^ T cell. *B*, naïve murine CD4^+^ T cells were activated for 4 days with constant anti-CD3, soluble anti-CD28 antibodies, and TGF-β with variable doses of IL-6. Cells were stained for CD4, FoxP3, and IL-17a. Flow cytometry was utilized where the abundance of IL-17a and FoxP3 was measured in CD4^+^ T cells across three independent experiments. *C*, the percent of CD4^+^ T cells that were FoxP3+ or IL-17a+ from the flow cytometry experiments presented in panel *B* was plotted as a function of IL-6 dose. *D*, cell lysates from murine CD4^+^ T cells were activated for 10 min with constant anti-CD3, anti-CD28 antibodies, and TGF-β with varying amounts of IL-6. Immunoblotting for p-STAT3(Y705), STAT3, p-SMAD3 (S423/425), and SMAD3 was performed. Densitometry was performed on immunoblots from panel *D* where (*E*) p-STAT3(Y705) was normalized to total STAT3. Shown are mean ± SD. *F*, primary murine CD4^+^ T cells were activated under Th17 or Treg polarization conditions for 10 min in the presence or absence of the JAK2 inhibitor. Cell lysates were immunoblotted for p-STAT (Y705), total STAT3, p-SMAD3 (S423/425), and SMAD3. Densitometry was performed on immunoblots across three biological replicates to determine the abundance of (*G*) p-STAT3 (Y705) normalized to total STAT3 abundance. Shown are mean ± SD; *p* values calculated by one-way ANOVA. Murine CD4^+^ T cells isolated by negative selection were activated for 10 min with constant anti-CD3, anti-CD28, and TGF-β with varying amounts of IL-6. *H*, SMAD3 was immunoprecipitated and the IPs were immunoblotted for SMAD3, SMAD4, and STAT3. *I*, densitometry was performed on immunoblots from panel *H* normalized to SMAD3 in the IP. Each datapoint represents the mean ± standard deviation across three independent experiments. Primary murine CD4^+^ T cells were activated under Th17 or under Treg polarization conditions for 10 min ± JAK2 inhibitor (200 nM) and SMAD3 was Immunoprecipitated. *J*, SMAD3 immunoprecipitates were immunoblotted for SMAD3, SMAD4, and STAT3. Densitometry was performed on immunoblots across three biological replicates for (*K*) STAT3 and (*L*) SMAD4 normalized to SMAD3. Shown are mean ± SD; *p* values were calculated by one-way ANOVA. Source data are provided for the immunoblots in panels *D*, *F*, *H*, and *J*.

Our previous work demonstrated that TGF-β receptor signaling promoted a negative feedback loop that downmodulated TCR and PI3K signaling during Treg induction (44). In this loop, the TGF-β receptor phosphorylated SMAD3 yielding the SMAD3/4 trimer (Fig. 5*A*). Work from other groups in HaCaT cells demonstrated that phosphorylated STAT3 downstream of IL-6 receptor signaling bound SMAD3. The formation of the STAT3-SMAD3 complex disrupted canonical TGF-β receptor signaling (66). Possibly, phosphorylated STAT3 outcompetes SMAD4 for binding SMAD3 reducing SMAD3/4 abundance during Th17 differentiation (Fig. 5*A*). To calibrate our *in vitro* activation system, naïve murine CD4^+^ T cells were activated with constant TCR, CD28, and TGF-β stimulation, while the concentration of IL-6 in the culture was varied (Fig. 5*B*). At low IL-6 doses, CD4^+^FoxP3^+^ T cells were abundant and decreased with higher IL-6 concentrations (Fig. 5, *B* and *C*). Conversely, few CD4^+^Il-17a^+^ T cells were present at low IL-6 and increased with Il-6 concentration (Fig. 5, *B* and *C*).

We first sought to characterize how the strength of the IL-6 receptor signal modulated STAT3 phosphorylation. The abundance of p-STAT3(Y505) increased as a function of IL-6 in T cells activated with constant TCR and TGF-β stimulation (Fig. 5, *D* and *E*). IL-6 receptor signal strength did not impact p-SMAD3(Y423/425) abundance (Fig. 5*D*). IL-6 receptor signals through Janus Kinases (JAKs) to phosphorylate Y705 on STAT3. Inhibition of JAK2 with a small molecule inhibitor reduced phosphorylation of Y705 of STAT3 under Th17 polarization conditions (Fig. 5, *F* and *G*), while the abundance of p-SMAD3(S423/425) was not impacted (Fig. 5*F*).

CD4^+^ T cells were activated with constant TCR/CD28 and TGF-β signals with varying doses of IL-6 to determine if IL-6 receptor signal strength regulates STAT3-SMAD3 or SMAD3-SMAD4 complex abundance. The condition containing 0 ng/ml of IL-6 is the condition utilized for Treg polarization, while the condition with 50 ng/ml IL-6 induces Th17 polarization. At low levels of IL-6, SMAD3 coprecipitated with SMAD4 (Fig. 5, *H* and *I*). With increased IL-6, the amount of SMAD4 coprecipitating with SMAD3 decreased. Little STAT3 coprecipitated with SMAD3 at low IL-6 doses (Fig. 5, *H* and *I*). The abundance of STAT3/SMAD3 increased as a function of IL-6 receptor signal strength and became the dominant complex at high IL-6 doses (Fig. 5, *H* and *I*). To determine if JAK2 phosphorylation of STAT3 was required for the formation of the STAT3-SMAD3 complex, primary CD4^+^ T cells activated under Th17 or Treg activation conditions in the presence or absence of the JAK2 inhibitor, and SMAD3 was immunoprecipitated. Immunoblotting revealed that blocking STAT3 phosphorylation disrupted the STAT3-SMAD3 interaction under Th17 activation conditions (Fig. 5, *J* and *K*). Additionally, blocking STAT3 phosphorylation by JAK2 inhibition increased SMAD3/4 complex abundance to levels observed during Treg polarization (Fig. 5, *J* and *L*).

### IL-6 signaling reduces PKA activation during Th17 polarization

**Figure 6:**
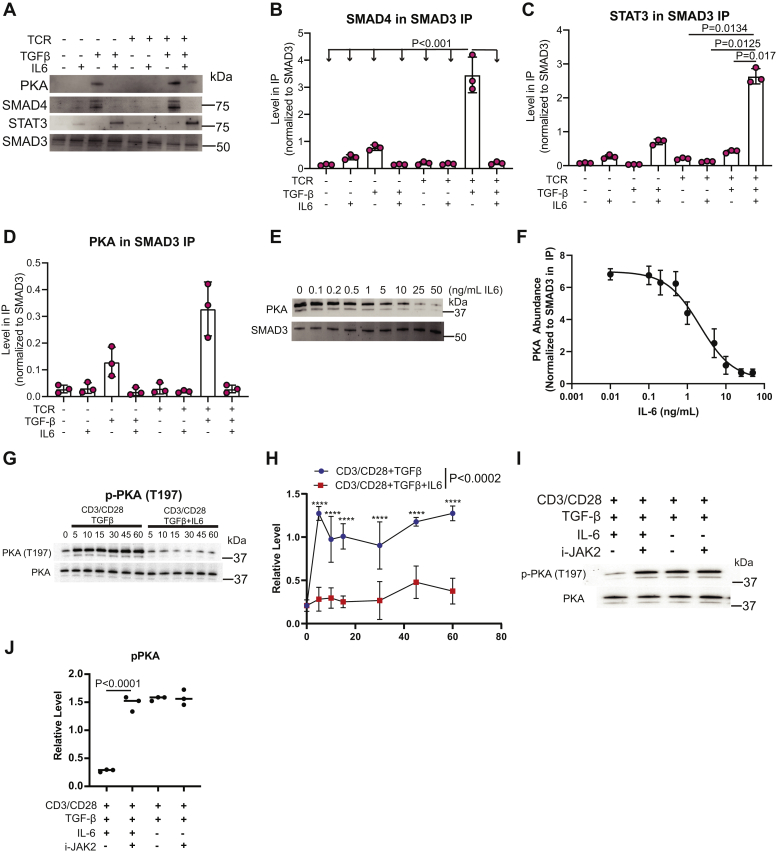
**IL-6 receptor signaling blocks SMAD3/4 activation of PKA during Th17 induction.***A*, primary murine CD4^+^ T cells isolated by negative selection were activated with different combinations of TCR/CD28, TGF-β, and IL-6 signaling inputs for 10 min. SMAD3 was immunoprecipitated. Immunoblotting was performed for SMAD3, STAT3, SMAD4, and PKA. Densitometry was performed on immunoblots to determine the relative amount of (*B*) SMAD4, (*C*) STAT3, and (*D*) PKA in the SMAD3 IP across three biological replicates. Shown are mean ± SD; *p* values were calculated by one-way ANOVA. *E*, primary murine CD4^+^ T cells were activated for 10 min with constant amounts of plate bound anti-CD3 antibody, soluble anti-CD28 antibody, and TGF-β with varying amounts of IL-6. SMAD3 was immunoprecipitated and immunoblotting was performed to monitor the amount of PKA that coprecipitated with SMAD3. This experiment corresponds to the data presented in Figure 5*B* and the SMAD3 immunoblots are therefore identical between both panels. *F*, densitometry was performed on immunoblots from panel *F* across three biological replicates where PKA was normalized to SMAD3 in the IP. *G*, primary murine CD4^+^ T cells activated under Treg (plate bound anti-CD3 antibody, soluble anti-CD28 antibody, and TGF-β) or Th17 (plate bound anti-CD3 antibody, soluble anti-CD28 antibody, TGF-β, and IL-6) stimulation for various time points. Immunoblotting for p-PKA (T197) and total PKA protein were performed. *H*, densitometry was performed on immunoblots from panel *G* across three biological replicates. Shown are mean ± SD; *p* values were calculated by two-way ANOVA (∗∗∗∗*p* < 0.0001). *I*, CD4^+^ T cells were activated under TH17 or Treg conditions for 10 min in the presence or absence of 200 nM JAK2 inhibitor. Cell lysates were immunoblotted for p-PKA (T197) and total PKA protein. *J*, densitometry was performed on immunoblots from panel *I* where p-PKA (T197) was normalized to total PKA protein across three independent experiments. Shown are mean ± SD; *p* values were calculated by one-way ANOVA. Source data are provided for the immunoblots in panels *A*, *E*, *G* and *I*.

Murine CD4^+^ T cells were activated with combinations of TCR/CD28, TGF-β, and IL-6 to define further how these signaling inputs regulated SMAD3 complex formation. SMAD4 coprecipitated with SMAD3 in T cells activated with TCR+TGF-β and at a lower level with just TGF-β signaling alone (Fig. 6, *A* and *B*). Focusing on STAT3, TGF-β+IL-6 promoted a moderate amount of SMAD3/STAT3 complex, and stimulation with TCR/CD28+TGF-β+IL-6 signals promoted the robust formation of the SMAD3/STAT3 complex (Fig. 6, *A* and *C*). Our previous work demonstrated that the SMAD3/4 trimer bound and activated PKA during the early phase of Treg induction (44). The TGF-β stimulation alone resulted in PKA coprecipitation with SMAD3 (Fig. 6, *A* and *C*). TCR+TGF-β stimulation resulted in maximum PKA coprecipitation with SMAD3 (Fig. 6, *A* and *D*). These data demonstrated that CD4^+^ T cells utilize SMAD3 interaction networks to decode TCR/CD28, TGF-β, and IL-6R signals.

Primary murine CD4^+^ T cells were activated with constant TCR and TGF-βR simulation, while the amount of IL-6 in the culture was varied to test the prediction that IL-6 signaling modulated SMAD3 binding to PKA. Immunoblotting revealed that IL-6 receptor signaling reduced the abundance of the SMAD3-PKA complex in a dose-dependent manner (Fig. 6, *E* and *F*). SMAD3/4 binding activated PKA to autophosphorylate itself on T197 (Fig. 6, *G* and *H*). Primary murine CD4^+^ T cells were activated under Treg or Th17 conditions in the presence or absence of JAK2 inhibitor. This analysis revealed that JAK2 inhibition increased p-PKA (T197) under Th17 conditions to levels observed in Treg activation conditions (Fig. 6, *I* and *J*). These data were consistent with a model where SMAD3/4 binding enhanced PKA activation under Treg conditions and the IL-6 signal in Th17 induction downmodulated PKA activation by disrupting SMAD3/4 formation.

### IL-6 blocks TGF-βR suppression of TCR and PI3K signaling in murine CD4^+^ T cells

**Figure 7:**
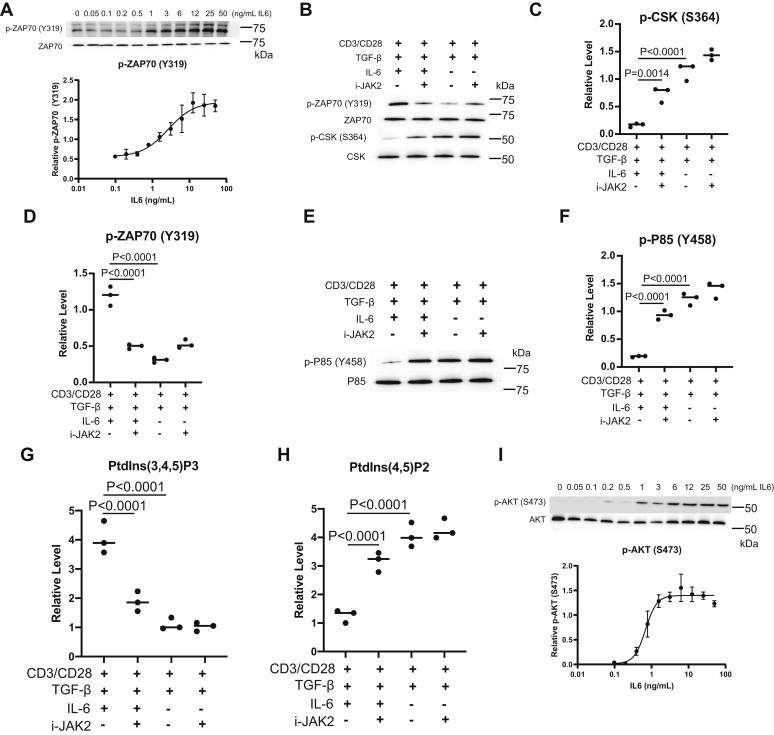
**JAK2 inhibition dysregulates signaling networks during Th17 polarization.***A*, primary murine CD4^+^ T cells were activated for 10 min with constant amounts of anti-CD3 antibody, soluble anti-CD28 antibody, and TGF-β with varying amounts of IL-6. Immunoblotting was performed for p-ZAP7(Y319) and total ZAP70. Densitometry was performed on immunoblots across three biological replicates. Shown are mean ± SD. Primary murine CD4^+^ T cells were activated under Th17 (TCR+TGF-β+IL-6) or Treg polarization conditions (TCR+TGF-β) for 10 min in the presence or absence of 200 nM JAK2 inhibitor. *B*, immunoblotting was performed for p-ZAP7(Y319), ZAP70, p-CSK-S364, and CSK. Densitometry was performed on immunoblots across three biological replicates from panel *B* to determine the relative amount of (*C*) p-CSK-S364 and (*D*) p-ZAP70(Y319) normalized to total CSK and Zap70 abundance respectively. Shown are mean ± SD; *p* values were calculated by one-way ANOVA. *E*, primary murine CD4^+^ T cells were activated under Th17 (TCR+ TGF-β+IL-6) or Treg polarization conditions (TCR+ TGF-β) for 10 min in the presence or absence of 200 nM JAK2 inhibitor. Immunoblotting was performed for p-P85 (Y458) and P85. *F*, densitometry was performed on immunoblots across three biological replicates from panel *E* were p-P85(Y458) was normalized to total P85. Shown are mean ± SD; *p* values were calculated by one-way ANOVA. Mass ELISA assays were utilized to determine the relative level of (*G*) PtdIns(3,4,5)P3, and (*H*) PtdIns(4,5)P2 generated in primary murine CD4^+^ T cells activated under Th17 or Treg polarization conditions for 10 min in the presence or absence of the JAK2 inhibitor. Shown are mean ± SD from three biological replicates; *p* values were calculated by one-way ANOVA. *I*, primary murine CD4^+^ T cells were activated for 10 min with constant amounts of anti-CD3 antibody, anti-CD28 antibody, and TGF-β with varying amounts of IL-6. Immunoblotting was performed for p-AKT(S473) and total AKT. Densitometry was performed on immunoblots across three biological replicates. Shown are mean ± SD. Source data are provided for the immunoblots in panels *A*, *B*, *E*, and *I*.

In our model of TCR-IL-6-TGF-β signaling (Fig. 5*A*), the IL-6 receptor phosphorylates STAT3 to promote the formation of a STAT3-SMAD3 complex to block SMAD3/SMAD4 complex formation. In blocking SMAD3/4 assembly, IL-6 receptor signaling diminished PKA activation. The prediction is that IL-6 would promote elevated TCR signaling. To test the model, primary murine T cells were activated with constant TCR/CD28 and TGF-β and varying doses of IL-6. This analysis demonstrated that the abundance of p-ZAP70 (Y319) increased with the strength of the IL-6 receptor signal (Fig. 7*A*).

IL-6 receptor-mediated formation of the STAT3-SMAD3 complex depended on JAK2 catalyzed formation of STAT3 (Fig. 5, *F* and *G*). The prediction is that JAK2 inhibition would suppress TCR signaling by promoting CSK activation in T cells activated under Th17 polarization. Murine CD4^+^ T cells were activated under Th17 and Treg conditions in the presence or absence of a JAK2 inhibitor. Immunoblotting revealed that JAK2 inhibition increased the abundance of p-CSK (S364) under Th17 conditions (Fig. 7, *B* and *C*). JAK2 inhibition also reduced p-ZAP70 (Y319) abundance during Th17 induction to levels observed during Treg polarization (Fig. 7, *B* and *D*). These data supported a model where IL-6 receptor signaling functioned to maintain elevated TCR signaling during Th17 polarization.

The prediction is that JAK2 inhibition would suppress PI3K signaling by promoting CSK activation in T cells activated under Th17 polarization. JAK2 inhibition increased the abundance of p-P85 (Y458) in Th17 polarization conditions to levels observed in Treg polarization (Fig. 7, *E* and *F*). Phosphorylation of P85 correlates to disruption of the P85-P110 heterodimer in CD4^+^ T cells. Consistent with increased p-P85(Y458), JAK2 inhibition reduced the abundance of PtdIns(3,4,5)P3 (Fig. 7*G*) and increased the abundance of PtdIns(4,5)P2 under Th17 induction conditions (Fig. 7*H*).

MTORC2 catalyzed phosphorylation of S473 on AKT requires high PtdIns(3,4,5)P3 in CD4^+^ T cells (33). Th17 polarization generated high PtdIns(3,4,5)P3 levels, while Treg polarization generated low levels. To characterize the threshold for IL-6 receptor signal strength needed to restore p-AKT (S473) abundance, primary murine T cells were activated with constant TCR/CD28 and TGF-β and varying doses of IL-6, which demonstrated a sharp IL-6 threshold for promoting p-AKT (473) (Fig. 7*I*). These data revealed JAK2 activity is necessary for IL-6R signaling to block TGF-βR suppression of TCR and PI3K signaling.

## Discussion

Herein, we utilized a phosphoproteomic approach to interrogate how CD4^+^ T cells integrate signals from the T cell, TGF-β, and IL-6 receptors that control the Treg *versus* Th17 cell fate decision. This analysis revealed that both additive and synergistic interactions between TCR, TGF-βR, and IL-6R signaling shaped the phosphoproteomes that differentially regulated pathways, including TCR proximal signaling, phosphatidylinositol metabolism, and AKT/mTOR signaling (Fig. 1). This work revealed that CD4^+^ T cells integrate T cell, TGF-β, and IL-6 receptor signals by generating different SMAD3 complexes (Fig. 5). In the context of cell fate choices, IL-6R signaling during Th17 polarization potentiated the formation of a SMAD3/STAT3 complex, whereas Treg polarization generated the SMAD3/4 heterotrimer. In turn, many signaling pathways, including TCR (Fig. 2) and PI3K (Fig. 3), were dampened *via* a SMAD3/4-PKA-CSK inhibitory circuit during Treg induction (Figs. 6 and 7). However, the flux through TCR and PI3K pathways was maintained during Th17 polarization because the SMAD3/STAT3 complex did not engage the PKA-CSK inhibitory circuit. Together, this work defined at the molecular level how T cells integrate signaling inputs from multiple receptors to promote the Treg *versus* Th17 cell fate choice.

SMAD3 has well-established transcriptional functions in T cells. There is growing evidence that SMAD3 complexes function to regulate processes besides transcription, including metabolic networks and kinase signaling. TGF-β derived from tumors suppresses the ability of CD4^+^ T cells to produce IFN-y, limiting their effector functions against tumors (67). Mechanistically, TGF-β promoted SMAD translocation to the mitochondria and inhibited ATP synthase activity by inhibiting mitochondrial complex V. These metabolic alterations imposed by mitochondrial SMADs impaired IFN-y production by CD4^+^ T cells. We previously reported that TGF-βR signaling promoted the formation of SMAD3/4, which bound and activated PKA, independent of cAMP, to suppress both TCR and PI3K signal strengths to levels optimal to induce Treg differentiation (44). Our proteomic analysis further demonstrated that TGF-βR signaling during Treg polarization suppressed signaling through the AKT/mTOR pathway by promoting inhibitory phosphorylation sites on mTOR and TSC2 (Fig. 4, *B* and *C*), which is consistent with previous reports that TGF-βR signaling inhibits CD4^+^ T cell activation through mTOR suppression (68). Here, we identified that IL-6R signaling promotes the phosphorylation of STAT3, and phosphorylated STAT3 competes with SMAD4 for binding SMAD3 during Th17 polarization (Fig. 5, *H* and *I*). At the concentration of Il-6 used for *in vitro* Th17 polarization assays, the STAT3-SMAD3 complex is abundant compared with SMAD3/4. By blocking the formation of SMAD3/4 *via* generation of STAT3/SMAD3, the IL-6R signal during Th17 induction functions to generate higher TCR and MTOR signaling levels, which is consistent with previous reports that a weak TCR signal favors Treg differentiation while a stronger TCR signal is required for Th17 differentiation (69, 70, 71). The role of SMAD3 on Treg differentiation is exemplified when SMAD3-deficient cells display a reduction in the Foxp3 expression (72), as well as SMAD3-deficient mice have a loss of homeostasis in the immune system and develop spontaneously a chronic inflammation (73). Thus, the complex SMAD3-4 plays a role on Treg cells and its disruption by IL-6 pathway leads to a reduction in the amount of Treg cells or in its suppressive capacity.

Most mechanistic knowledge of cytokine signaling in T cells is based on studies detailing signaling from individual cytokines and associated receptors. However, T cells function in complex environments where multiple cytokine signaling inputs must be integrated to generate context-dependent immune responses. Previous work demonstrates that the integration of IL-4 and IL-2 signals is not additive in Tregs (74). IL-4 stimulation of STAT6 phosphorylation is downmodulated by IL-2 signaling. There was a synergistic interaction between IL-2 and IL-4 signaling that increases STAT5 phosphorylation and IL-10 production, which contributes to increased Treg suppressive capacity both *in vitro* and *in vivo*. There are many possible mechanisms by which T cells could integrate cytokine signaling inputs. The simplest model would invoke additivity. In this model, the signaling events that occur in T cells activated simultaneously with multiple stimuli could simply be explained by adding together signals that occur during activation with individual signaling inputs. In the context of Treg differentiation, this would mean that the sum of the phosphoproteome observed from TCR/CD28 and TGF-β would be equivalent to the phosphoproteome observed with the combined TCR/CD28+TGF-β stimulus. Many phosphoproteins are shared between the TCR/CD28 or TGF-β stimuli and the combined TCR/CD28+TGF-β stimulation based on the phosphoproteomic data (Fig. 1). However, a significant portion of the TCR/CD28+TGF-β phosphoproteome cannot be explained by adding the individual TCR/CD28 or TGF-β stimuli (Fig. 1*E*). Generally, this pattern holds across the different combinations of signaling inputs tested. Adding the phosphoproteome generated by individual signaling inputs does not fully describe the phosphoproteome generated when a T cell receives multiple stimuli simultaneously. Invoking synergy between multiple signaling inputs is required. An example from the Treg *versus* Th17 cell fate choice is the formation of SMAD3 complexes (Fig. 6*A*). SMAD3/4 is weakly formed with TGF-β alone, and little SMAD3/4 is formed from just TCR/CD28. However, SMAD3/4 formation is high in response to combined TCR/CD28+TGF-β, which is not explained by summing the abundance of SMAD3/4 generated by the individual TCR/CD28 and TGF-β conditions. Downstream, SMAD3/4 binding to PKA also exhibited a synergistic interaction between TCR/CD28 and TGF-β signals (Fig. 7, *A* and *D*). In considering STAT3-SMAD3 complex formation, there is no combination of individual stimuli nor pairs of stimuli administered simultaneously that sums to generate the amount of STAT3-SMAD3 formed in response to TCR/CD28+TGF-β+IL-6 (Fig. 6, *A* and *C*). Together, our data demonstrate that both additive and synergistic interactions between TCR/CD28, TGF-βR, and IL-6R signals shape signaling networks that drive Treg and Th17 cell fate choices.

In conclusion, this work provides a biochemical basis for how a T cell interprets multiple receptor signaling inputs. Classically, receptor cross talk in T cells is described at the level of transcriptional regulation (75, 76). Our current work demonstrates that CD4^+^ T cells generate distinct phosphoproteomes in response to different combinations of TCR/CD28, IL-6, and TGF-β signaling inputs. TCR and cytokine signals are integrated by kinase signaling and protein interaction networks within minutes of activation to control pathways critical for the Treg *versus* Th17 cell fate choice, including AKT/mTOR and metabolic pathways. We posit that, in addition to initiating transcriptional programs, signals from cytokine receptors synergize to engage other biological pathways to promote T cell differentiation programs. This work establishes a conceptual framework for understanding how T cells integrate multiple signaling inputs to program cell fate decisions.

## Experimental procedures

### D10 cell line culture and activation

D10.G4.1 murine T cells were obtained (ATCC) and cultured in RPMI 1640 media (Thermo Fisher) supplemented with 2 mM L-glutamine, 100 units/ml penicillin, 10 μg/ml streptomycin, 55 μM 2-Mercaptoethanol (BME), 10% fetal bovine serum (Thermo Fisher), and 0.05 μg/ml recombinant mouse IL-2 (BioLegend). The cells grew in a humidified incubator at 37 °C with 5% CO_2_ and 95% air to a density of approximately 1 × 10^6^ cells/ml before activation. The cells were rested in the above media without BME or IL-2 for 2 h in the incubator prior to activation. To activate cells, 2 × 10 (7) cells per sample were at a density of 1 × 10^6^ cells/ml with 100 ng of IL-2. Different samples were also incubated with varying combinations of the following: 200 ng recombinant TGF-β1 (BioLegend), 1 μg recombinant IL-6, and 500 μl mouse T-activator CD3/CD28 Dynabeads (Thermo Fisher). Cells were activated for 10 min in the incubator, then removed, and immediately placed on ice to stop the activation.

### Sample preparation and phosphopeptide enrichment

Activated D10.G4.1 Murine CD4^+^ T cells were lysed in a buffer containing 10% SDS, 50 mM triethylammonium bicarbonate (TEAB) (Sigma Aldrich) pH 7.55, Complete C protease (Roche), and PhosSTOP phosphatase inhibitors (Roche). The resulting lysates were sonicated and centrifuged to clear lysate from any insoluble debris. In total, 300 μg of protein from the above lysate was reduced using 20 mM DTT (Sigma Aldrich) for 10 min at 95 °C. Protein alkylation was performed with 40 mM iodoacetamide (Sigma Aldrich) for 30 min. Samples were acidified by adding 12% phosphoric acid to a final concentration of 1.2% and then diluted sevenfold with 90% methanol in 100 mM TEAB. Samples were then added to an S-trap mini column (ProtiFi) and washed three times in 90% methanol in 100 mM TEAB. Trypsin (Promega) was added at a ratio of 1:25 trypsin/protein in a solution of 50 mM TEAB pH 7.1 and allowed to digest overnight at 37 °C. Peptides were then eluted in three buffers 80 μl 50 mM TEAB pH 7.1, 80 μl of 0.2% formic acid, and 80 μl 50% acetonitrile and 0.2% formic acid. The three elutions were combined and desalted using C18 columns (Supelco) on a vacuum manifold and dried in a vacuum centrifuge. For phosphopeptide enrichment, samples were resuspended in 80% acetonitrile and 0.1% trifluoroacetic acid. Samples were processed on an AssayMAP Bravo protein sample preparation platform (Agilent). Phosphopeptides were enriched using the Fe-NTA(III) cartridges (Agilent) and eluted using in a buffer of 20 μl of 1% ammonia.

### Label free LC-MS analysis of D10 activation

Phosphopeptides were resolved with liquid chromatography–tandem mass spectrometry using a system composed of a Waters nanoACQUITY UPLC in-line with a Q-Exactive mass spectrometer (Thermo Fisher). Samples were run in a randomized order. Solvent A (0.1% formic acid in water, Burdick & Jackson) and solvent B (0.1% formic acid in acetonitrile, Burdick & Jackson) were used as the mobile phase. Peptides were then eluted from a capillary column (100 μm inner diameter × 100 mm long; ACQUITY UPLC M-Class Peptide BEH C18 Column, 1.7-μm particle size, 300 Å (Waters) and resolved using a 100-min gradient at a flow rate of 0.9 μl/min (4–33% B for 90 min, 33–80% B for 2 min, constant at 80% B for 6 min, and then 80–0% B for 2 min to equilibrate the column). Data were collected in positive ionization mode.

PEAKS Studio 10.0 build 20190129 was used to sequence and identify peptides. The UNIProt_SwissProt: *Mus musculus* (house mouse) sequence database was used, which contained 17013 entries. One missed cleavage by trypsin was permitted in the database search. Carbamidomethylation was treated as a fixed modification. Acetylation (Protein N-term), oxidation (M), and phosphorylation (S, T or Y) were set as variable modifications. The mass tolerance for precursor ions was set to 20 ppm, and mass tolerance for fragment ions set to 0.8 Da. The FDR for peptide/spectrum matches is reported as 9.5%, calculated by decoy fusion. Label-free quantitation was performed using the quantitative module in the PEAKSX software. Individual values for all quantitative mass spectrometric measurements are depicted and the mean peak area is depicted as a bar graph. Error bars represent ± the standard deviation. *p* values were calculated with one-way ANOVA to assess statistical significance.

### Biological pathway analysis

The following criteria were used to identify proteins that were differentially phosphorylated between activation conditions: (1) a phosphopeptide peptide had to be identified by the mass spectrometric analysis; and (2) its abundance had to be more than twofold greater based on the mass spectrometric label-free quantitation to be assigned as specific to a group. The Ingenuity software package was used to perform a statistical overrepresentation test to identify pathways differentially targeted by phosphorylation between the activation groups.

### Primary murine CD4^+^ T cell isolation and activation

Mice were housed at the University of Pittsburgh in a pathogen-free facility and handled under Institutional Animal Care and Use Committee–approved guidelines. A negative selection kit (Miltenyi Biotech) was utilized to isolate CD4^+^ T cells from C57BL/6 spleens. T cells were rested for 1 h at 37 °C. T cells were activated using plates that were coated with a 3 μg/ml of anti-CD3 monoclonal antibody (clone 17A2 BioLegend) and 1 μg/ml soluble anti-CD28 mAb (Clone 37.51 BioLegend). Variable doses of recombinant IL-6 and TGF-β1 (BioLegend). For experiments using the small-molecule JAK2 inhibitor (JAK2 Inhibitor IV, CAS# 1110502-30-1, Calbiochem), CD4^+^ T cells were incubated with 200 nM for 1 h prior to activation.

### Western blotting

PAGE was performed using BioRad precast Protein TGX gels. Proteins were transferred to PVDF membranes using a BioRad Trans-Blot Turbo transfer system with the preprogrammed mixed molecular weight setting. Antibodies used for Western blotting purchased from Cell Signaling Technology included:

p-ERK1/2(T202/204)(D13.14.4E), ERK1/2 (137F5), CSK (C74C1), p-ZAP70(Y319) (65E4), ZAP70 (D1C10E), p-P85(Y458), (E3U1H), P85 (19H8), P110 (D1Q7R), p-AKT(S473) (D9E), AKT (C67E7), p-S6 (S235/236)(D57.2.2E), S6 (5G10), STAT3 (124H6), SMAD3 (C67H9), p-PKA(T197) (D45D3) and PKA (D38C6). Antibodies used for Western blotting purchased from Thermo Fisher included: p-CSK(S364) (PA5-40214). Antibodies used for Western blotting purchased from Abcam included: p-STAT3 (Y705), p-SMAD3 (S423,S425) (EP823Y). All primary antibodies utilized were rabbit. An anti-Rabbit IgG-HRP antibody (Cell Signaling Technology 7074) was used with the SuperSignal West Femto chemiluminescent substrate for detection on either a protein simple FluorChem M or a BioRad ChemiDoc system. Densitometry quantitation was performed with the ImageJ software package.

### Immunoprecipitation of P85 or PKA

Murine CD4^+^ T cells were activated for 10 min. Cells were lysed in a buffer containing 1% NP40, 50 mM Tris (pH = 8.0), 150 mM NaCl, and Complete C phosphatase inhibitor cocktail. Lysates were incubated at room temperature for 2 h with antibodies specific for either P85 (Cell Signaling Technology, 19H8) or PKA (Cell Signaling Technology, D38C6). Magnetic protein A beads (Cell Signaling Technology) were utilized to capture the immune complexes, which were analyzed by Western Blotting as described above.

### Imaging flow cytometric measurement of phosphatidylinositol abundance

Murine CD4^+^ T cells isolated by negative selection were activated. Following activation, cells were incubated in one volume of 2× Cytofix/Perm/Wash Buffer (3%PFA + BD Biosciences Perm/Wash Buffer at room temperature for 15 min and on ice for 30 min. Cells were washed with Perm/Wash buffer. Cells were stained with antibodies against TCR-APC (BD Biosciences clone H57–597) and CD4-PerCP-5.5 (BD Biosciences clone RM4-5). Cells were also stained with antibodies against PtdIns(4,5)-PE (Echelon Biosciences Z-B045) or PtdIns(3,4,5)P3-PE (Echelon Biosciences Z-B3345B). Cells were washed with Perm/Wash buffer and resuspended in PBS containing 3% Fetal serum, 2 mM EDTA, 0.02% sodium azide. Analysis was performed on an AMNIS Image Stream MarkII imaging flow cytometer. Cells were gated on the TCR^+^CD4^+^ population. Analysis was performed with the IDEAS software package.

### CD4^+^ T cell proliferation and differentiation

Naive CD4+CD25-CD44 low T cells were purified from lymph nodes and spleens of wild-type C57BL/6 mice using a CD4^+^ T cell isolation kit (Miltenyi Biotech) and an AutoMACS magnetic cell sorter (Miltenyi Biotech) according to the manufacturer's protocol. Purified cells were activated with plate coated anti-CD3 and soluble anti-CD28 (anti-CD3: 4 μg/ml; anti-CD28: 2 μg/ml BD Biosciences) on flat-bottom plates (1 × 105/well) for Th17 cells or coated with anti-CD3 and anti-CD28 (both 2 μg/ml; BD Biosciences) on flat-bottom plates (1 × 105/well) for Treg cells. Skewing conditions were as follows: Th17: 2.5 ng/ml rhTGF-β1 (Thermo Scientific) plus 20 ng/ml rmIL-6 (R&D Systems); iTreg polarization: 3 ng/ml rhTGF-β1 (Thermo Scientific). After isolation, the cells were stained with a cell trace (Carboxylic Acid Diacetate, Succinimidyl Ester (Carboxy-DFFDA, SE, CAS# C34555, ThermoScientific) for 15 min at 37 °C in incomplete medium (IMDM, ThermoScientific) and 5% CO_2_. Then, the cells were washed with complete medium and incubated at same conditions. The cells were cultured under Th17 or Treg conditions for 4 days. For the intracellular staining, cells were stimulated with PMA (Phorbol 12-myristate 13-acetate), ionomycin (Sigma-Aldrich), and Golgi stop (BD Biosciences) for 4 h and then stained for viability, CD4, IL-17A and/or FoxP3 with transcription factor staining buffer set (CAS# 00-5523-00, ThermoScientific). The cells were analyzed on a LSR II cell analyzer (BD Biosciences) and data was processed using FlowJo v10.7.1 (BD Biosciences).

### Mass ELISA assay for measuring PtdIns(4,5)P2 and PtdIns(3,4,5)P3

Murine CD4^+^ T cells isolated by negative selection were activated. Pellets from 2 million cells were washed with ice cold 0.5 M TCA and treated with 750 μl of MeOH: CHCl_3_: 12N HCl (80:40:1), vortexing for 30 min and centrifuging for 10 min at 3000 RPM to remove neutral lipids. The supernatant was treated with 250 μl of CHCl3 and 450 μl of 0.1 N HCl. The organic phase was dried under a stream of nitrogen gas and reconstituted in PBS. Mass ELISA kits from Echelon Biosciences were used to measure PtdIns(3,4,5)P3 and PtdIns(4,5)P2 using the manufacturer’s protocol using a Molecular Devices SpectraMax i3 plate reader.

## Data availability

The raw mass spectrometric data was deposited to the PRIDE database (https://www.ebi.ac.uk/pride/) under the accession number PXD025506.

## Supporting information

This article contains supporting information.

## Conflict of interest

The authors declare that they have no conflicts of interest with the contents of this article.
