# Supplementary material for: Synergistic and additive interactions between receptor signaling networks drive the regulatory T cell versus T helper 17 cell fate choice
Source: J Biol Chem. 2021 Oct 21;297(6):101330. doi: 10.1016/j.jbc.2021.101330 (PMC8645459; doi:10.1016/j.jbc.2021.101330)
Supplement: Figures S1–S3 [file mmc1.pdf]

Supporting information

Synergistic and additive interactions between receptor signaling networks drive the regulatory T cell versus T helper 17 cell fate choice

Douglas S. Prado[1, ‡] , Richard T. Cattley[1, ‡], Corey W. Shipman[1], Cassandra Happe[2], Mijoon Lee[3], William C. Boggess[3], Matthew L. MacDonald[2], William F. Hawse[1,*]

Material included

Fig. S1 Overview of the proteomic experimental approach, phosphoproteomic data and analysis.

Fig. S2 Label free quantitation of the phosphoproteomic data for the T cell receptor signaling pathway.

Fig. S3 Label free quantitation of the phosphoproteomic data for phosphatidylinositol metabolism.

Table S1 Raw phosphoproteomic data.

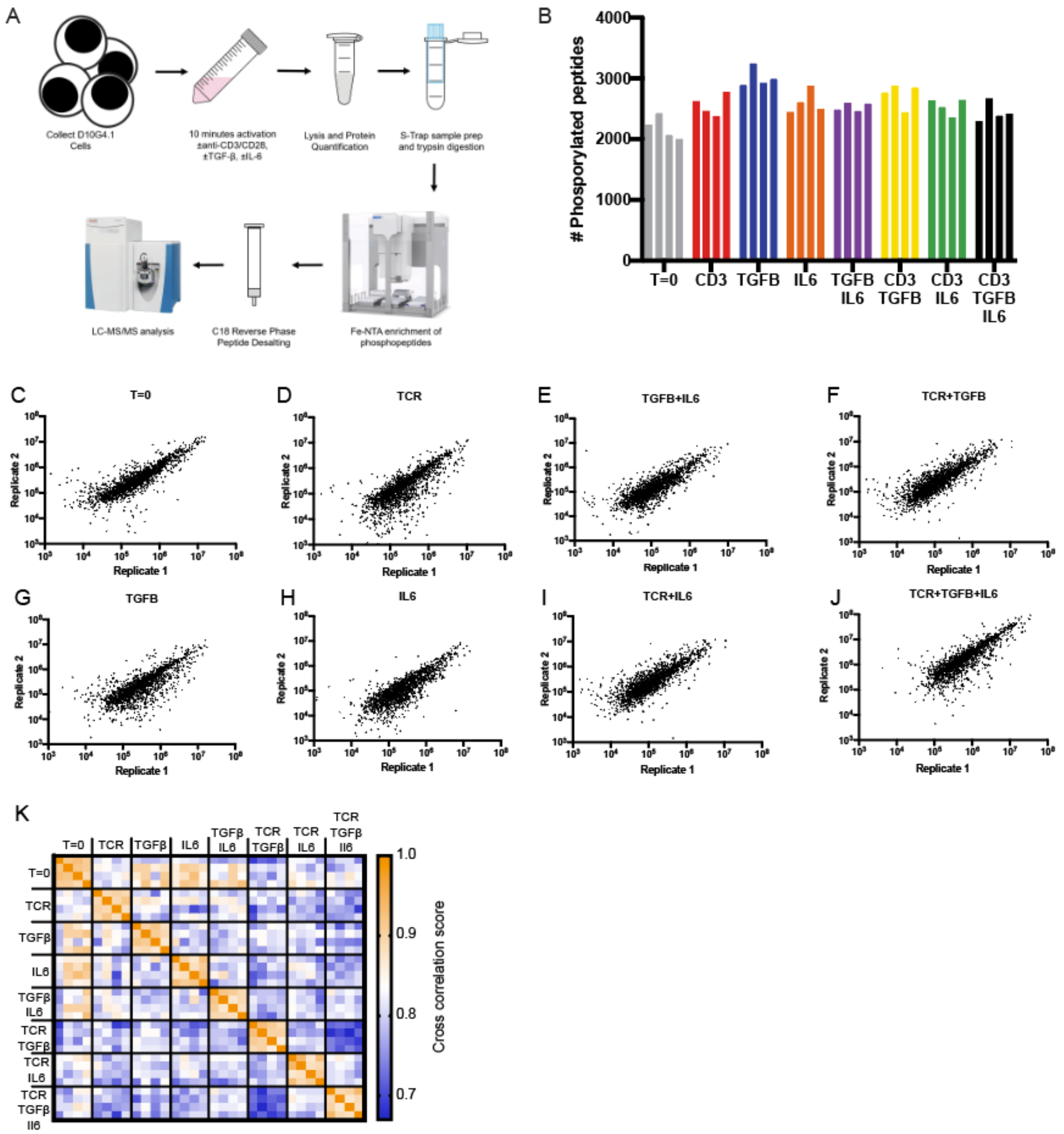

Fig. S1 overview of the proteomic experimental approach, phosphoproteomic data and analysis.

(**A**) D10 CD4+ T cells were activated with different combinations of CD3/CD28, TGF-β and IL6 for ten minutes at 37 °C. Cells were lysed, protein levels were quantified and normalized across samples and an S-trap based tryptic digestion was utilized to generate peptides. Fe-NTA enrichment was performed to isolate phosphopeptides, which were desalted using C18 columns. Samples were analyzed by mass spectrometry. The resulting data was processed in the PEAKs software package to obtain relative quantitation for each phosphopeptide over four biological replicates. (**B**) The number of phosphorylated peptides identified by mass spectrometry for each biological replicate are depicted. (**C-J**) The integrated peak area derived from label free quantitation for each identified phosphopeptide was plotted for two samples from each stimulation condition. (**K**) The cross-correlation matrix based on label free phosphoproteomic analysis was constructed, where each square represents the level of correlation between each experimental sample analyzed.

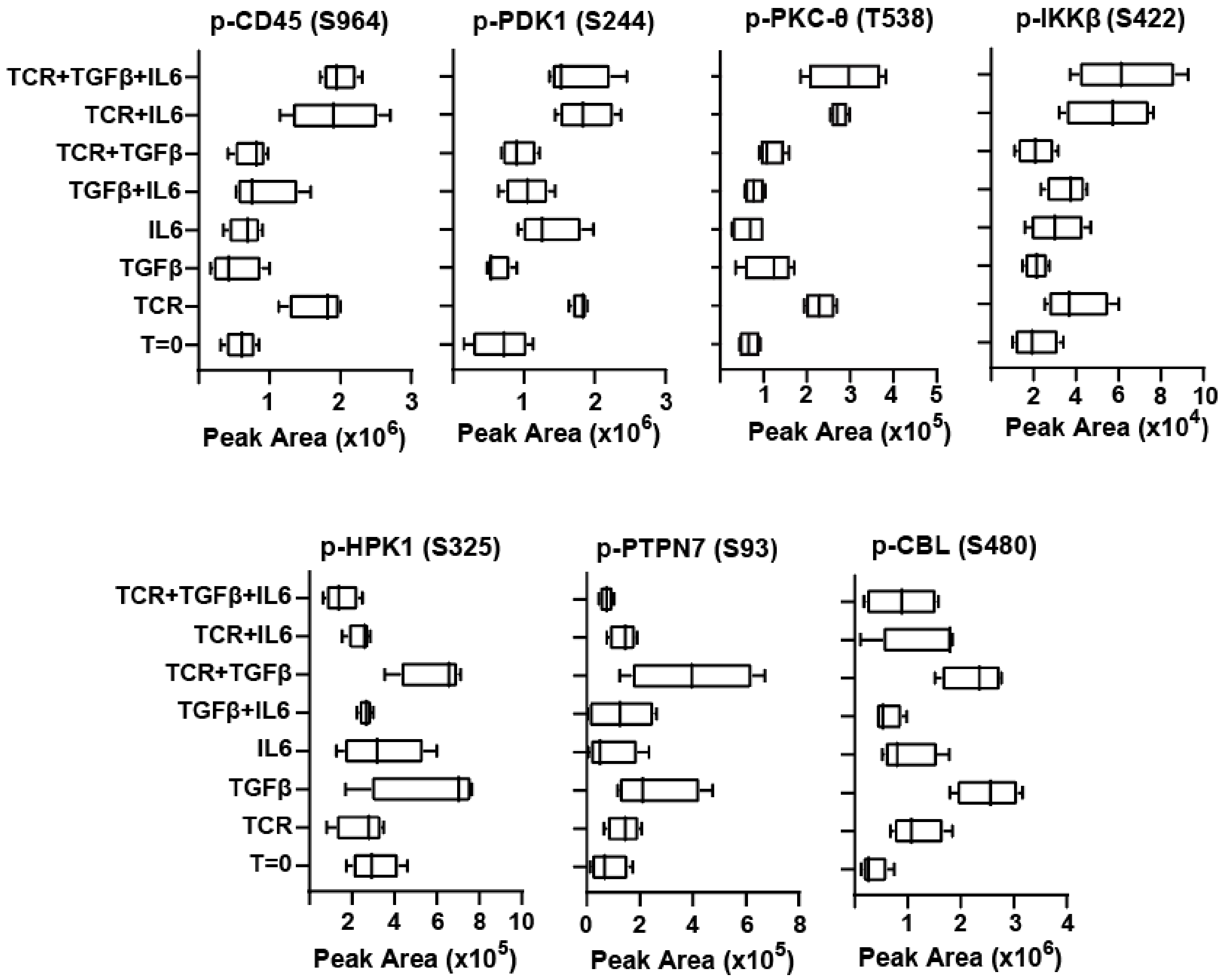

Fig. S2 Label Free quantitation of the phosphoproteomic data for the T cell signaling pathway.

The label-free phosphoproteomic analysis determined the relative abundance of phosphopeptides for kinases and phosphatases regulated by signaling input in the T cell receptor signaling pathway. The peak area defined by the label-free quantitation is depicted for phosphorylation sites on CD45, PDK1, PKC, IKKB, HPK1, PTPN7 and CBL across the various stimulation conditions. In the bar and whiskers plot, the bottom of the bar represents the 25th percentile, the top represents the 75th percentile, and the line within the box represents the mean across four independent experiments. The whiskers are drawn the lowest and highest peak areas observed.

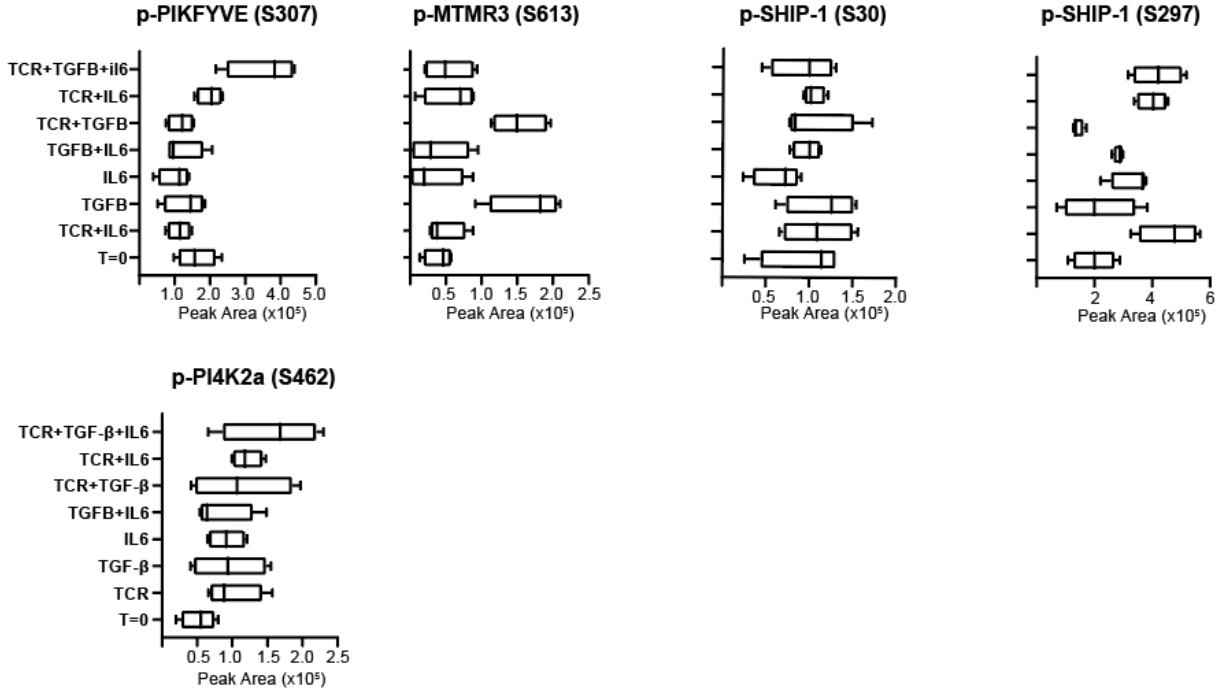

Fig S3. Label-free quantitation of the phosphoproteomic data for phosphatidylinositol metabolism.

The label-free phosphoproteomic analysis determined the relative abundance of phosphorylation on lipid kinases and phosphatases regulated by signaling input. In the bar and whiskers plot, the bottom of the bar represents the 25th percentile, the top represents the 75th percentile, and the line within the box represents the mean across four independent experiments. The whiskers are drawn the lowest and highest peak areas observed.
